# Zero-fluoroscopy, intracardiac echocardiography–only patent foramen ovale closure in early pregnancy: a case report

**DOI:** 10.1093/ehjcr/ytag635

**Published:** 2026-09-02

**Authors:** SungHo Jo, Tae-Hoon Kim, Yong-Joon Lee, KyeongHo Yun, Jung-Sun Kim

**Affiliations:** Department of Cardiology, Wonkwang University Hospital, 895 Muwang-ro, Iksan, Jeonbuk 54538, Republic of Korea; Division of Cardiology, Severance Hospital, Yonsei University College of Medicine, 50-1 Yonsei-ro, Seodaemun-gu, Seoul 03722, Republic of Korea; Division of Cardiology, Severance Hospital, Yonsei University College of Medicine, 50-1 Yonsei-ro, Seodaemun-gu, Seoul 03722, Republic of Korea; Department of Cardiology, Wonkwang University Hospital, 895 Muwang-ro, Iksan, Jeonbuk 54538, Republic of Korea; Division of Cardiology, Severance Hospital, Yonsei University College of Medicine, 50-1 Yonsei-ro, Seodaemun-gu, Seoul 03722, Republic of Korea

**Keywords:** Patent foramen ovale, Pregnancy, Ischaemic stroke, Intracardiac echocardiography, Zero-fluoroscopy, Case-report

## Abstract

**Background:**

Transcatheter closure of a patent foramen ovale (PFO) is an established strategy for secondary prevention of cryptogenic stroke in selected patients. During pregnancy, the timing and method of closure require individualized assessment of recurrent embolic risk and maternal–fetal procedural risk. In selected first-trimester patients, avoiding fluoroscopy, contrast, procedural transoesophageal echocardiography (TEE), and sedation may be desirable when adequate alternative imaging is available.

**Case summary:**

A 34-year-old woman at 7 weeks’ gestation presented with acute ischaemic stroke. Work-up demonstrated a non-lacunar cortical infarct, long-tunnel PFO with a large provoked right-to-left shunt, and no alternative embolic source; the Risk of Paradoxical Embolism score was 9. After multidisciplinary discussion, early percutaneous closure was performed using a completely zero-fluoroscopy, intracardiac echocardiography (ICE)-only workflow under local anaesthesia, without procedural TEE. Key adaptations included use of a pre-shaped transseptal sheath for stable cannulation and a left atrial rail using a pre-shaped support wire. Stop-flow balloon sizing was performed and agitated saline was injected through the delivery sheath as an adjunctive visual check of left atrial position prior to device release. A 25/18-mm Amplatzer Talisman PFO occluder was deployed successfully with no complications.

**Discussion:**

This case demonstrated the feasibility of zero-fluoroscopy, zero-contrast PFO closure under ICE-only guidance in a carefully selected first-trimester pregnant patient. The combination of stable sheath engagement, a pre-shaped left atrial support-wire rail, stop-flow balloon sizing, and sheath-through agitated saline injection facilitated controlled device deployment without fluoroscopy or contrast. This approach depends on operator expertise and institutional familiarity with ICE-guided structural intervention.

Learning points

- In pregnant patients with embolic stroke and high-risk PFO features, closure timing should be individualized through multidisciplinary assessment and patient preference.
- Zero-fluoroscopy, zero-contrast PFO closure under ICE guidance may be feasible in selected patients when performed by operators experienced in ICE-guided structural intervention.
- In an ICE-only workflow, sheath-through agitated saline injection may complement ICE assessment of left atrial position before device release.

## Introduction

Transcatheter closure of a patent foramen ovale (PFO) is an established therapy for prevention of recurrent embolic stroke in selected patients.^1,2^ Pregnancy adds complexity because randomized PFO closure trials did not specifically address pregnant patients, and both recurrent embolic risk and maternal–foetal procedural risk must be considered. Although fluoroscopic procedures can generally be performed during pregnancy with very low foetal exposure using radiation-minimization strategies, avoiding fluoroscopy, iodinated contrast, procedural transoesophageal echocardiography (TEE), and additional sedation may be desirable in selected first-trimester patients. Intracardiac echocardiography provides real-time visualization of atrial septal anatomy, sheath position, and device deployment during structural heart interventions.^3^ We report a zero-fluoroscopy, zero-contrast, intracardiac echocardiography (ICE)-guided PFO closure in a woman at 7 weeks’ gestation, focusing on the clinical decision-making and procedural safeguards used in this selected setting.

## Summary figure

**Figure:**
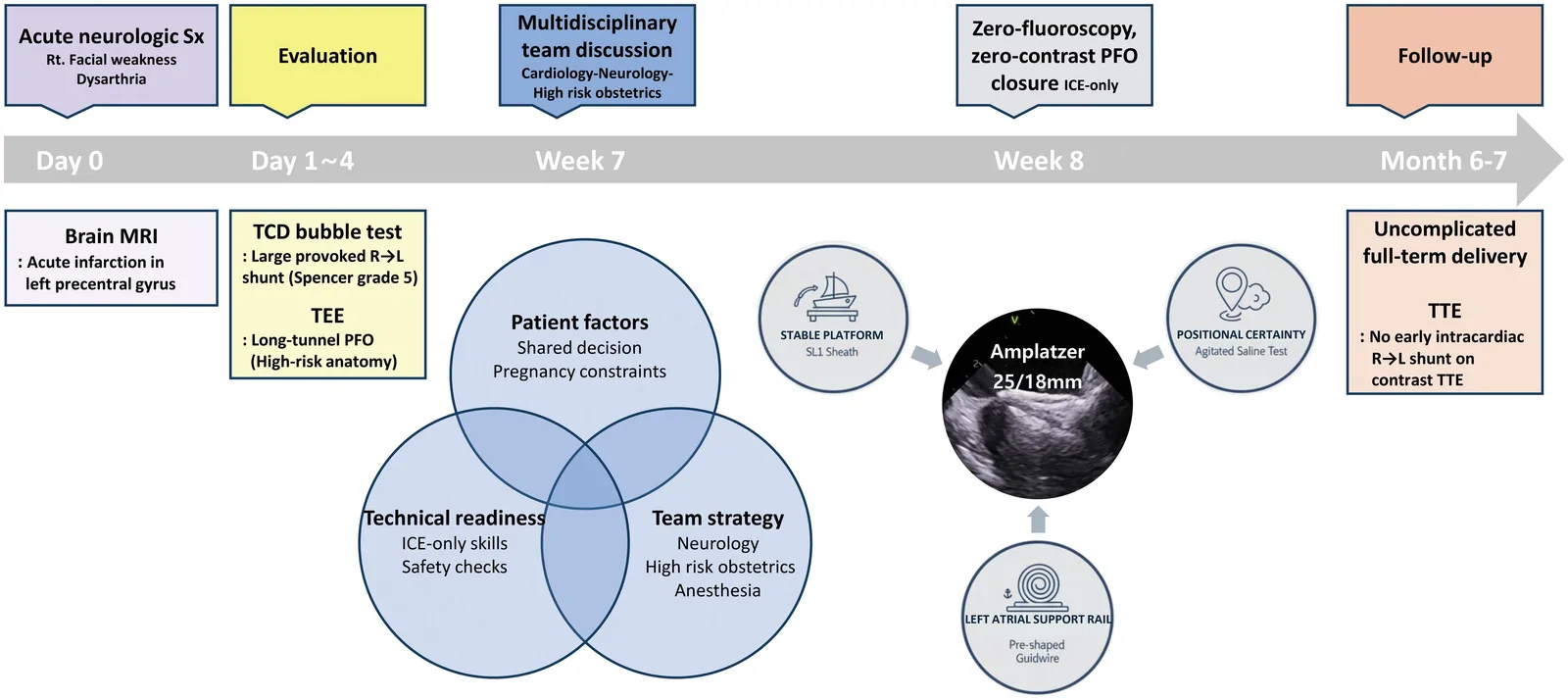


## Case presentation

**Figure 1:**
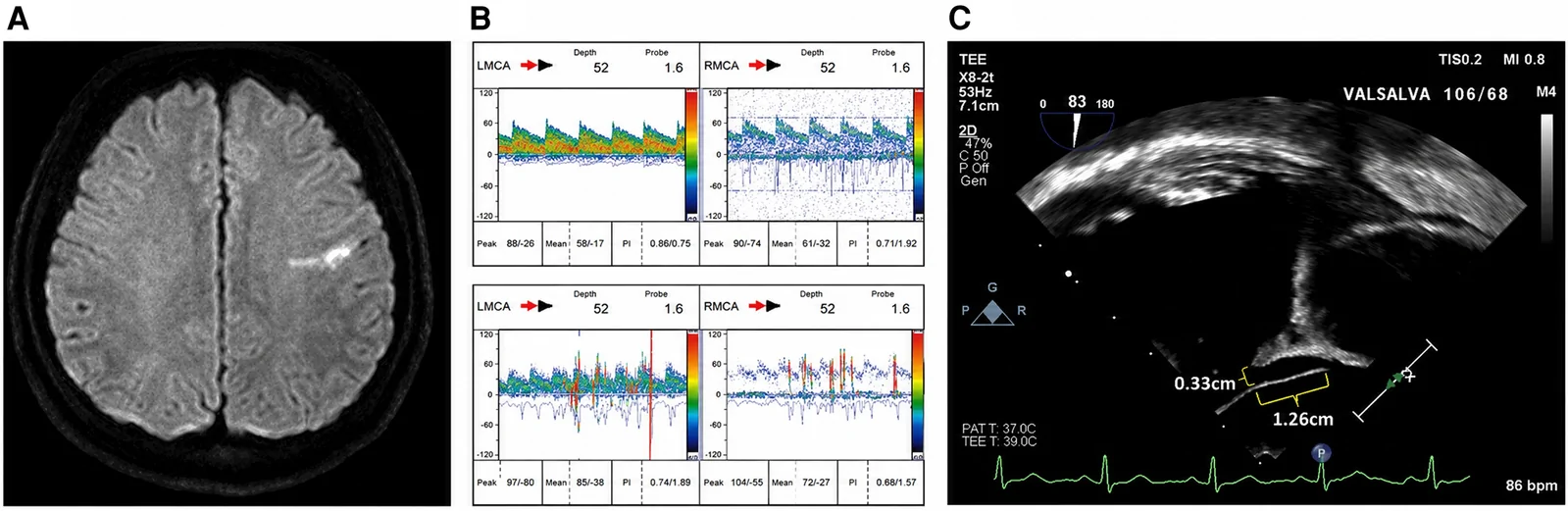
Diagnostic imaging. (*A*) Brain MRI demonstrating an acute infarction in the left precentral gyrus. (*B*) Transcranial Doppler bubble study with provocation demonstrating numerous high-intensity transient signals, consistent with a large right-to-left shunt. (*C*) Transoesophageal echocardiography demonstrating a long-tunnel patent foramen ovale.

A 34-year-old woman (G2P1) at 7 weeks’ gestation presented with acute-onset right facial droop and dysarthria. Brain magnetic resonance imaging (MRI) demonstrated an acute non-lacunar infarction in the left precentral gyrus. She had no traditional vascular risk factors. Transcranial Doppler with agitated saline demonstrated a large provoked right-to-left shunt (Spencer grade 5), and TEE showed a long-tunnel PFO (*Figure 1*). The Risk of Paradoxical Embolism score was 9, supporting a likely PFO-associated index stroke. No alternative embolic source was identified after systematic evaluation, including rhythm monitoring, lower-limb venous ultrasonography, and thrombophilia/antiphospholipid antibody testing.

A multidisciplinary team comprising interventional cardiology, neurology, and maternal–foetal medicine considered medical therapy with delayed postpartum closure vs. early percutaneous closure. After shared decision-making, early closure was selected based on the risk of recurrent embolism during pregnancy, maternal–foetal procedural considerations, and the patient’s preference to avoid prolonged anticoagulation.

**Figure 2:**
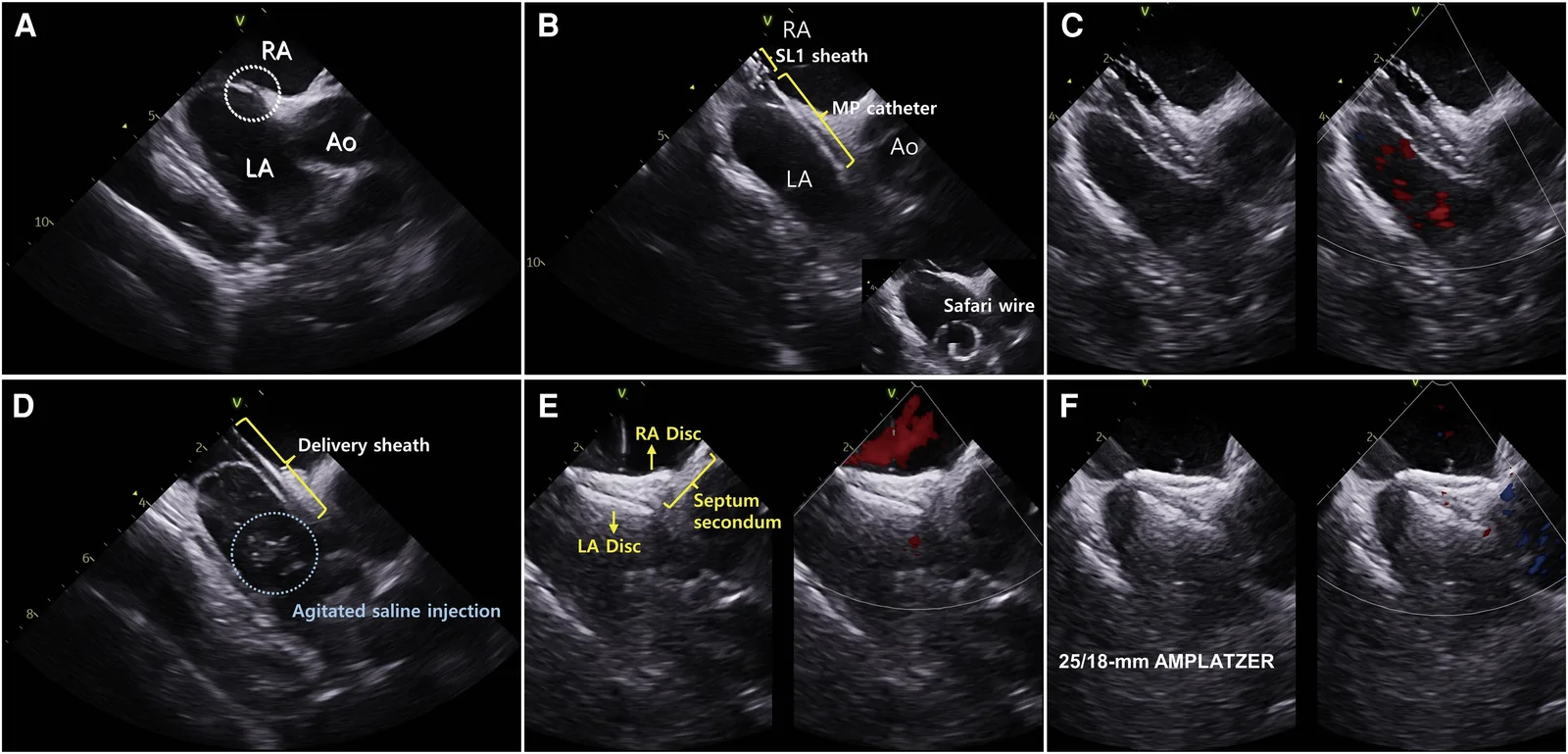
Zero-fluoroscopy, intracardiac echocardiography-only patent foramen ovale closure workflow. (*A*) Initial intracardiac echocardiography examination delineating the patent foramen ovale tunnel. (*B*) Pre-shaped transseptal sheath positioned at the fossa ovalis; inset shows a Safari support wire forming a pre-shaped left atrial rail. (*C*) Stop-flow balloon sizing demonstrating an 11-mm stop-flow diameter. (*D*) Sheath-through agitated saline injection showing dense left atrial microbubble opacification, used as an adjunctive visual check of left atrial position before device release. (*E*) Intracardiac echocardiography confirms left and right atrial discs straddling the septum. (*F*) Final intracardiac echocardiography colour Doppler demonstrates no residual shunt.

## Procedure

Under local anaesthesia, femoral venous access was obtained. Intravenous unfractionated heparin was administered after venous access, targeting an activated clotting time of 250–300 s. ICE imaging (ACUNAV; Siemens) was used throughout the procedure. A pre-shaped transseptal sheath (SL1; Abbott) was positioned against the fossa ovalis to provide a stable platform. Through the sheath, the PFO was traversed using a multipurpose catheter and a hydrophilic wire. The hydrophilic wire was exchanged for a pre-shaped support wire (Safari; Boston Scientific), whose pre-formed loop was positioned within the left atrial cavity under ICE guidance to provide a support rail while avoiding deep pulmonary vein cannulation. Stop-flow balloon sizing demonstrated an 11-mm stop-flow diameter, and a 25/18-mm Amplatzer Talisman PFO occluder (Abbott) was selected based on the stop-flow diameter and long-tunnel anatomy. The SL1 sheath was then exchanged for the device delivery sheath. Sheath-through agitated saline injection showed dense left atrial microbubble opacification, which was used as an adjunctive visual check of left atrial position before device release (*Figure 2*).

The device was deployed successfully. Final ICE imaging with colour Doppler confirmed stable device apposition and no residual shunt.

## Follow-up

The patient was discharged on post-procedure day 1 on dual antiplatelet therapy with aspirin 100 mg once daily and clopidogrel 75 mg once daily for 2 months, followed by aspirin monotherapy (100 mg once daily) according to institutional practice. She had an uncomplicated full-term delivery approximately 6 months after the procedure. At 7 months, transthoracic echocardiography with agitated saline contrast showed fewer than 10 bubbles only after 8–9 cardiac cycles, with no early intracardiac right-to-left shunt, consistent with successful PFO closure. Aspirin monotherapy was continued, and no recurrent neurologic or thromboembolic event occurred.

## Discussion

Evidence regarding the optimal timing of PFO closure during pregnancy is limited, and pregnancy-specific guidance is based largely on expert consensus.^4^ Prior reports suggest that, in selected patients, antepartum closure can be performed while minimizing foetal procedural exposure: Daehnert *et al*. used TEE guidance without fluoroscopy after recurrent strokes, whereas Schrale *et al*. used ICE with minimal radiation in three second-trimester cases.^5,6^ These reports demonstrate the feasibility of exposure-sparing antepartum closure in selected patients. In our patient, presentation at 7 weeks’ gestation meant that deferral would leave the interatrial conduit present throughout most of pregnancy and the peripartum period. Her non-lacunar cortical infarct, RoPE score of 9, Spencer grade 5 shunt, long-tunnel anatomy, and the absence of another embolic source supported a clinically important PFO-associated stroke. After multidisciplinary shared decision-making, early closure was selected because an experienced team could perform an ICE-only, zero-fluoroscopy, zero-contrast procedure under local anaesthesia without procedural TEE.

Conventional PFO closure is usually performed with fluoroscopic guidance, often supported by TEE or ICE. Fluoroscopy is not absolutely contraindicated during pregnancy when appropriate radiation-minimization strategies are applied.^7^ In this patient, ICE provided continuous visualization of septal anatomy, sheath position, and device deployment, allowing successful closure without fluoroscopy, contrast, or procedural TEE.

The procedural contribution of this case was the integration of stable sheath engagement, a pre-shaped left atrial support-wire rail, stop-flow balloon sizing, and sheath-through agitated saline injection within an ICE-only workflow. The saline injection complemented ICE assessment of left atrial position before device release.

This single-case experience is dependent on operator and centre expertise and may not be applicable to all PFO anatomies. Longer-term follow-up remains warranted to assess late device-related events and recurrent neurological outcomes.

## Conclusion

Zero-fluoroscopy, zero-contrast PFO closure under ICE-only guidance was feasible in this carefully selected first-trimester pregnant patient.

## Lead author biography


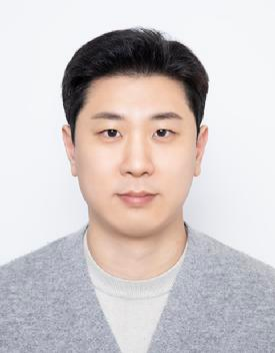


Sungho Jo, MD, is an Assistant Professor of Cardiology at Wonkwang University Hospital, Iksan, Republic of Korea. His clinical and research interests include structural heart disease interventions, intracardiac echocardiography-guided procedures, and complex coronary interventions with a focus on chronic total occlusion. He is particularly interested in developing reproducible low- or zero-radiation workflows and imaging-based strategies to improve procedural safety in special clinical situations, including pregnancy.

## Supplementary Material

ytag635_Supplementary_Data

## Data Availability

All data relevant to the case are included in the article and its supplementary material.

## References

[1] Kavinsky CJ, Szerlip M, Goldsweig AM, Amin Z, Boudoulas KD, Carroll JD, et al SCAI guidelines for the management of patent foramen ovale. J Soc Cardiovasc Angiogr Interv 2022;1:100039. 39131947 10.1016/j.jscai.2022.100039 PMC11307505

[2] Kleindorfer DO, Towfighi A, Chaturvedi S, Cockroft KM, Gutierrez J, Lombardi-Hill D, et al 2021 guideline for the prevention of stroke in patients with stroke and transient ischemic attack: a guideline from the American Heart Association/American Stroke Association. Stroke 2021;52:e364–e467. 34024117 10.1161/STR.0000000000000375

[3] Alkhouli M, Hijazi ZM, Holmes DR, Rihal CS, Wiegers SE. Intracardiac echocardiography in structural heart disease interventions. JACC Cardiovasc Interv 2018;11:2133–2147. 30409271 10.1016/j.jcin.2018.06.056

[4] Swartz RH, Ladhani NNN, Foley N, Nerenberg K, Bal S, Barrett J, et al Canadian stroke best practice consensus statement: secondary stroke prevention during pregnancy. Int J Stroke 2018;13:406–419. 29171360 10.1177/1747493017743801

[5] Daehnert I, Ewert P, Berger F, Lange PE. Echocardiographically guided closure of a patent foramen ovale during pregnancy after recurrent strokes. J Interv Cardiol 2001;14:191–192. 12053303 10.1111/j.1540-8183.2001.tb00733.x

[6] Schrale RG, Ormerod J, Ormerod OJM. Percutaneous device closure of the patent foramen ovale during pregnancy. Catheter Cardiovasc Interv 2007;69:579–583. 17295329 10.1002/ccd.21031

[7] American College of Obstetricians and Gynecologists . Committee Opinion No. 723: guidelines for diagnostic imaging during pregnancy and lactation. Obstet Gynecol 2017;130:e210–e216. 28937575 10.1097/AOG.0000000000002355

